# Alterations in neuronal morphology and synaptophysin expression in the rat brain as a result of changes in dietary n-6: n-3 fatty acid ratios

**DOI:** 10.1186/1476-511X-12-113

**Published:** 2013-07-26

**Authors:** Toktam Hajjar, Yong Meng Goh, Mohamed Ali Rajion, Sharmili Vidyadaran, Tan Ai Li, Mahdi Ebrahimi

**Affiliations:** 1 Department of Veterinary Preclinical Sciences, Universiti Putra Malaysia, 43400 UPM, Serdang, Selangor, Malaysia; 2 Institute of Tropical Agriculture, Universiti Putra Malaysia, 43400 UPM, Serdang, Selangor, Malaysia; 3 Department of Pathology, Faculty of Medicine and Health Sciences, Universiti Putra Malaysia, Serdang, Selangor, Malaysia

**Keywords:** n-3 polyunsaturated fatty acid, Neuronal morphology, Synaptophysin, Hippocampus, Rats

## Abstract

**Background:**

Polyunsaturated fatty acids (PUFA) play important roles in brain fatty acid composition and behavior through their effects on neuronal properties and gene expression. The hippocampus plays an important role in the formation of memory, especially spatial memory and navigation. This study was conducted to examine the effects of PUFA and specifically different dietary n-6: n-3 fatty acid ratios (FAR) on the number and size of hippocampal neurons and the expression of synaptophysin protein in the hippocampus of rats.

**Methods:**

Forty 3-week old male *Sprague–Dawley* rats were allotted into 4 groups. The animals received experimental diets with different n-6: n-3 FAR of either 65:1, 26.5:1, 22:1 or 4.5:1 for 14 weeks.

**Results:**

The results showed that a lowering dietary n-6: n-3 FAR supplementation can increase the number and size of neurons. Moreover, lowering the dietary n-6: n-3 FAR led to an increase in the expression of the pre-synaptic protein synaptophysin in the CA1 hippocampal subregion of the rat brain.

**Conclusions:**

These findings support the notion that decreasing the dietary n-6: n-3 FAR will lead to an intensified hippocampal synaptophysin expression and increased neuron size and proliferation in the rat brain.

## Background

The hippocampus is one of the main regions of the brain which monitors learning and memory processing [1]. It is also responsible for spatial memory and plays important roles in cognition. Memory and learning are first acquired by specialized CA1 cells located in the hippocampal region, processed by CA2 cells which are responsible for the long term potentiation and retention of memories. The CA3 cells in turn are responsible for memory plasticity and re-learning abilities [2]. It is thus justified to conclude that the CA1, CA2 and CA3 subregions are crucial in spatial memory [3]. Spatial memory is important to animals as it enables them to locate foods, mates and defends their territories which are crucial for species survival [4]. The adult hippocampus undergoes many types of plasticity including neurogenesis [5], alteration in the morphology of the cells [6] and changes in synaptic strength [7]. Functions of the hippocampus such as learning and memory rely on this plasticity [8]. Different expressions of proteins involved in neurotransmission at the synapses are considered as the markers of neural plasticity. Synaptophysin is a presynaptic membrane protein essential for neurotransmission in hippocampal neurons [9]. It is one of the most widely used protein markers of synaptic plasticity in the brain [10,11]. Loss of this pre-synaptic vesicle protein in the hippocampus correlates with the cognitive decline in Alzheimer’s disease [11].

Studies have shown that PUFA supplementation is associated with an over-expression of synaptophysin in the hippocampus [12,13]. The n-3 PUFA incorporated into the neuron membrane increase synaptic protein expression, resulting in an increased dendritic spine formation, neurite outgrowth, synaptogenesis, and neurogenesis to strengthen the hippocampal synaptic plasticity and protect the neurons [14]. The n-3 PUFA play an important role in neurogenesis and neurite outgrowth, and also influences the neural membrane biogenesis [15]. Previous studies have shown that docosahexaenoic acid (22:6 n-3; DHA) enhanced neurite outgrowth of hippocampal and cortical neurons [16] and a deficiency of n-3 fatty acids decreases the cell body size of neurons in the hippocampus and hypothalamus, and decreases the complexity of dendritic arborizations on cortical neurons [17]. On the other hand, it has been shown that the larger perikaryal size is accompanied by an increased terminal density, and this combination is positively correlated with an improvement of memory [18]. Membrane function is highly dependent on membrane fluidity and integrity, which in turn are dependent on the lipid composition of the lipid bilayer [19]. Changes in the PUFA content of the neural membrane influence membrane fluidity, control the physiological functions of the brain, and also regulate synthesis and functions of brain neurotransmitters resulting in changes in synaptic plasticity and spatial cognition [20,21].

Fatty acids are crucial factors that determine the structure and function of biological membranes, including membranes in the nervous system. The brain has a higher lipid amount than any other organ in the body except adipose tissue. Neuronal membranes contain high concentrations of n-3 and n-6 PUFA [22]. Since dietary n-3 PUFA contribute to the construction and maintenance of the brain [23], they are also required for optimal cognitive performance [24]. In fact, our earlier report showed that rats fed with increasingly higher levels of n-3 PUFA vis-à-vis n-6 PUFA demonstrated marked improvement in cognitive, as well as spatial learning abilities [25]. These results pointed to the essentiality of n-3 PUFA in brain biochemistry, physiology, and functioning, and hence in cognitive performances during development [26]. There is a close interaction between PUFAs and the concentrations of various neurotransmitters in the brain that have relevance to long term potentiation (LTP) and memory formation [27]. A balance exists between various neurotransmitters in the brain, where a decrease in the levels of dopamine and serotonin may lead to an enhancement of the level of gamma-aminobutyric acid (GABA) and this, in turn, may contribute to learning deficits [27]. Several studies have shown that monoaminergic and cholinergic systems are influenced by chronically deprived n-3 PUFA during development in rodents [28]. In general, metabolism and function in the brain, especially neurotransmission, depend on preserving a homeostatically balanced concentration of n-3 and n-6 PUFA [29]. There is evidence to suggest that PUFA can enhance acetylcholine (ACH) release, which in turn may augment the events that facilitate memory and improvement of the learning ability [30], and prevent apoptosis of neurons [31]. The ACH is the principal vagal neurotransmitter and a known component of the parasympathetic nervous system [32]. This neurotransmitter modulates synaptic plasticity and LTP, which is a key component of memory consolidation, whereby the PUFA can improve the learning ability in rats [27].

The PUFAs also inhibit the production of the neurotoxic cytokine tumor necrosis factor (TNF), interleukin-1 (IL-1), and IL-6 and enhance nitric oxide (NO) synthesis, thereby preventing neuronal apoptosis and facilitate memory improvement and consolidation [27]. Thus, it is clear that PUFAs can enhance neuronal survival by protecting against the peroxidative damage of lipids and proteins in brains and attenuate neuron loss, resulting in an improved cognitive function [16]. Interestingly PUFA are also important natural agonists for Peroxisome Proliferator-Activated Receptors (PPAR). PPAR’s are subfamily members of the nuclear receptor super family, and are found extensively in neurons and microglials, two cell types that are crucial for neuronal remodeling [33]. PPAR’s have been reported to have pronounced neuroprotective and anti-inflammatory properties [34,35]. Therefore the upregulation of PPAR activities in the brain as a result of n-3 and n-6 supplementation reported in our earlier study [25], would have profound effects on the morphology of neurons. In view of the importance of n-3 PUFA to the central nervous system, particularly in the hippocampal region, this study was conducted to determine the impact of an increase of n-3 PUFA on the possible microscopic changes to the neuron morphology in the hippocampus. This is crucial as the retention of memory involves both chemical and morphological changes in the hippocampus we postulated that improved cognitive function that was reported earlier [25] may have been associated with both chemical and morphological changes brought upon by the PUFA supplementation in the hippocampus. These would have enabled us to correlate the potential positive outcome of neural plasticity as a result of n-3 PUFA supplementation, and n-3 PUFA’s potential roles in facilitating memory and learning. To address this objective, the size and number of neurons in the CA1 and CA3 hippocampus were analyzed. In this study, it was also determined whether different dietary n-6: n-3 FAR affected the hippocampal expression of synaptophysin, which is an essential factor for synaptic plasticity in the rat brain.

## Results

### Fatty acid profiles of the hippocampus

Brain Hippocampus fatty acid profiles as percentage of total fatty acids in the CTRL and fish oil and soybean oil supplemented groups are shown in Table 1.

**Table 1:** Fatty acid profile in rat hippocampus after 12 weeks of feeding trial (Mean ±1 SE)

	**Groups**			
**Fatty acid**	**CTRL**	**LMO**	**MMO**	**HMO**
Myristic Acid (14:0)	1.85 ± 0.14^a^	2.07 ± 0.26^a^	3.18 ± 0.07^b^	2.25 ± 0.44^ab^
Myristoleic Acid (14:1)	0.83 ± 0.11^b^	1.43 ± 0.30^b^	0.00 ± 0.00^a^	0.00 ± 0.00^a^
Palmitic Acid (16:0)	23.42 ± 0.79	22.40 ± 0.65	21.38 ± 0.59	22.27 ± 0.93
Stearic Acid (18:0)	22.77 ± 0.21	22.82 ± 0.35	22.23 ± 0.40	22.78 ± 0.64
Oleic Acid (18:1)	19.56 ± 0.34	19.10 ± 0.35	19.59 ± 1.28	20.17 ± 1.31
Linoleic Acid (18:2 n-6)	0.73 ± 0.10	0.92 ± 0.08	0.66 ± 0.11	0.81 ± 0.23
Linolenic Acid (18:3 n-3)	1.47 ± 0.11	1.81 ± 0.21	1.42 ± 0.41	1.44 ± 0.13
Nervonic Acid (24:1)	4.81 ± 0.14^ab^	4.86 ± 0.59^ab^	5.37 ± 0.30^b^	3.42 ± 0.57^a^
Arachidonic Acid (20:4 n-6)	10.91 ± 0.52	9.46 ± 0.51	9.28 ± 0.51	7.65 ± 0.32
Eicosapentaenoic acid (20:5n-3)	1.02 ± 0.21^b^	1.32 ± 0.34^b^	1.55 ± 0.43^ab^	2.45 ± 0.15^a^
Docosapentaenoic acid (22:5n-3)	0.13 ± 0.03	0.32 ± 0.02	0.26 ± 0.01	0.41 ± 0.04
Docosahexaenoic acid (22:6 n-3)	12.50 ± 0.62^a^	13.49 ± 0.70^ab^	13.95 ± 1.46^ab^	15.75 ± 0.38^b^
Total SFA	48.04 ± 0.71	47.30 ± 0.50	46.79 ± 0.46	47.30 ± 1.14
Total MUFA	25.20 ± 0.46	25.39 ± 1.02	24.96 ± 1.37	23.59 ± 1.49
Total n-3 PUFA	15.12 ± 0.66^a^	16.94 ± 0.51^ab^	17.18 ± 1.05^ab^	20.05 ± 0.27^b^
Total n-6 PUFA	11.64 ± 0.42	10.19 ± 0.44	10.01 ± 0.58	8.38 ± 0.49
Total PUFA	26.76 ± 1.03	27.13 ± 1.65	27.19 ± 1.98	28.43 ± 1.76
(n-6):(n-3)	0.77 ± 0.02^b^	0.60 ± 0.01^ab^	0.58 ± 0.05^ab^	0.42 ± 0.03^a^
UFA:SFA	1.09 ± 0.03	1.12 ± 0.02	1.14 ± 0.02	1.15 ± 0.05
PUFA:SFA	0.56 ± 0.03	0.57 ± 0.02	0.58 ± 0.03	0.60 ± 0.01

^a, b^ notations differ significantly within rows at P < 0.05.

*CTRL:* control group, *LMO:* low menhaden oil, *MMO:* medium menhaden oil and *HMO:* high menhaden oil. *SFA:* sum of C14:0, C16:0, C18:0. *MUFA:* sum of C14:1, C18:1, C24:1. Total n-3PUFA: sum of C18:3n-3, C20:5n-3, C22:5n-3, C22:6n-3. Total n-6PUFA: sum of C18:2n-6, C20:4n-6. (n-6): (n-3): Total n-6PUFA (C18:2n-6, C20:4n-6): Total n-3PUFA (C18:3n-3, C20:5n-3, C22:6n-3).

Supplementation of n-3 PUFA from fish oil significantly increased DHA content in the brain (P < 0.05) in HMO fed animals. The trend of the DHA concentration in the brain followed the order of HMO > MMO > LMO > CTRL (Table 1). These values were 15.75%, 13.95%, 13.49%, and 12.50% in HMO, MMO, LMO, and CTRL animals, respectively.

The amount of AA in the brain of CTRL animals was higher compared to the treatment groups (Table 1).

The fish oil fed animals had more total n-3 PUFA than CTRL groups in their brains (Table 1). A 7-fold increase in n-3 PUFA in MMO diet resulted in 13.62% increase in the brain n-3 PUFA. While in response to the 26-fold increase of n-3 PUFA diet, content of brain n-3 PUFA in HMO rats increased by 32.60% (*P* < 0.05).

The n-6 PUFA was lower in fish oil and soybean oil fed animals, the levels were in the decreasing order of LMO > MMO > HMO although they were not significantly different (Table 1). However, the fish oil and soybean oil fed animals had less n-6: n-3 fatty acid ratios in their brains when compared to CTRL groups. Based on Table 1, the HMO animals had the lowest n-6: n-3 fatty acid ratio, 0.42, which was significantly different (*P* < 0.05) from CTRL (0.77), MMO (0.55) and LMO (0.60) groups (Table 1).

### Size and number of neurons

Table 2 illustrates the size of the neurons in the CA1 layer of the hippocampus, which has an important role in spatial memory. In the Control (CTRL), low menhaden oil (LMO) and medium menhaden oil (MMO) groups, the CA1 neurons were significantly smaller compared to the neurons in the HMO group. The average size of neurons in the hippocampus CA1 in high menhaden oil (HMO) rats was 79.32 μm^2^ ± 3.83, while the area of these neurons in other groups ranged from 56.51- 59.97 μm^2^. Thus, the size of CA1 neurons was larger by 40% in the HMO group compared to the CTRL group (*P* < 0.05). The difference in neuron size between the MMO and LMO rats was not significantly different (*P* > 0.05) compared to the CTRL group (Table 2). The size of neurons in CA3 (89.91- 99.66 μm^2^) was larger than CA1 (56.51-79.32 μm^2^). There was no significant difference (*P* > 0.05) in the size of neurons in the CA3 layer in the LMO, MMO and HMO rats compared to CTRL rats.

**Table 2:** **Neuron size (μm**^**2**^**) in the hippocampus after 12 weeks of feeding trial (Mean ± SE)**

	**Group**			
**Hippocampal CA neurons**	**CTRL**	**LMO**	**MMO**	**HMO**
CA1	56.51 ± 2.42^a^	57.83 ± 2.01^a^	59.97 ± 2.69^a^	79.32 ± 3.83^b^
CA3	89.91 ± 3.09	85.56 ± 4.47	90.54 ± 4.25	99.66 ± 6.15

^a, b^ notations differ significantly within rows at *P <* 0.05.

*CTRL:* control group, *LMO:* low menhaden oil, *MMO:* medium menhaden oil and *HMO:* high menhaden oil.

**Figure 1:**
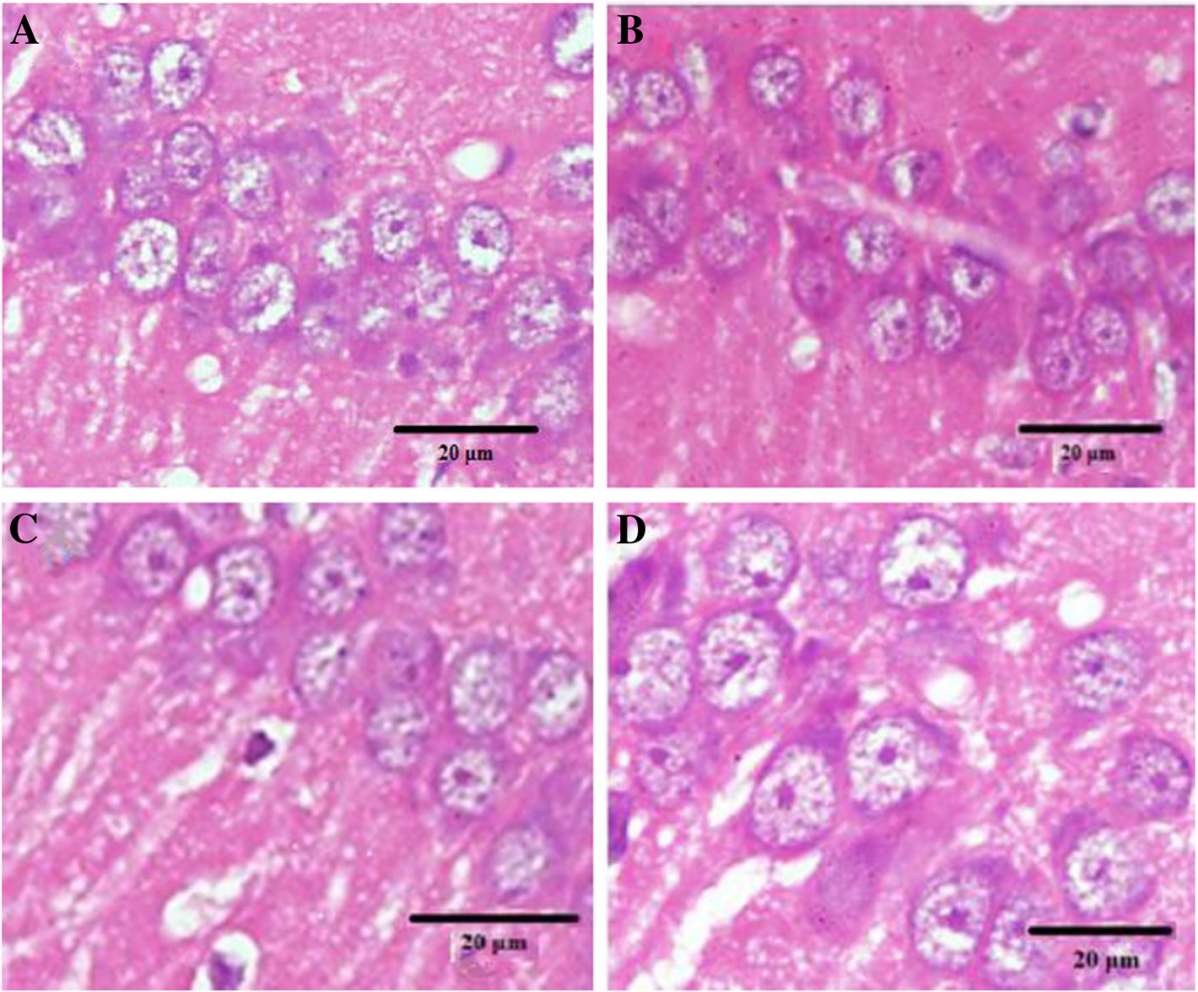
**Light micrograph of hippocampal CA1 neurons in rats.** CTRL **(A)**, LMO **(B)**, MMO **(C)** and HMO **(D)**. Note that the size of neurons in HMO rats **(D)** was larger than the other groups.

Table 3 illustrates the number of neurons in the hippocampus. The mean number of CA1 neurons of all supplemented groups was significantly higher than the CTRL group (*P* < 0.05). However, the numbers of neurons of the hippocampal CA3 region in the supplemented groups were not significantly different compared to CTRL rats. The light micrographs of the hippocampus from CTRL rats and supplemented groups are shown in Figure 1.

**Table 3:** Neuron number in the hippocampus after 12 weeks of feeding trial (Mean ± SE)

	**Group**			
**Hippocampal CA neurons**	**CTRL**	**LMO**	**MMO**	**HMO**
CA1	11.48 ± 0.38^a^	13.60 ± 0.43^b^	13.95 ± 0.90^b^	13.13 ± 1.07^b^
CA3	8.43 ± 0.39^a^	8.54 ± 0.38^a^	8.32 ± 0.21^a^	10.11 ± 1.10^a^

^a, b^ notations differ significantly within rows at P < 0.05.

*CTRL:* control group, *LMO:* low menhaden oil, *MMO:* medium menhaden oil and *HMO*: high menhaden oil.

### Synaptophysin immunohistochemistry

**Figure 2:**
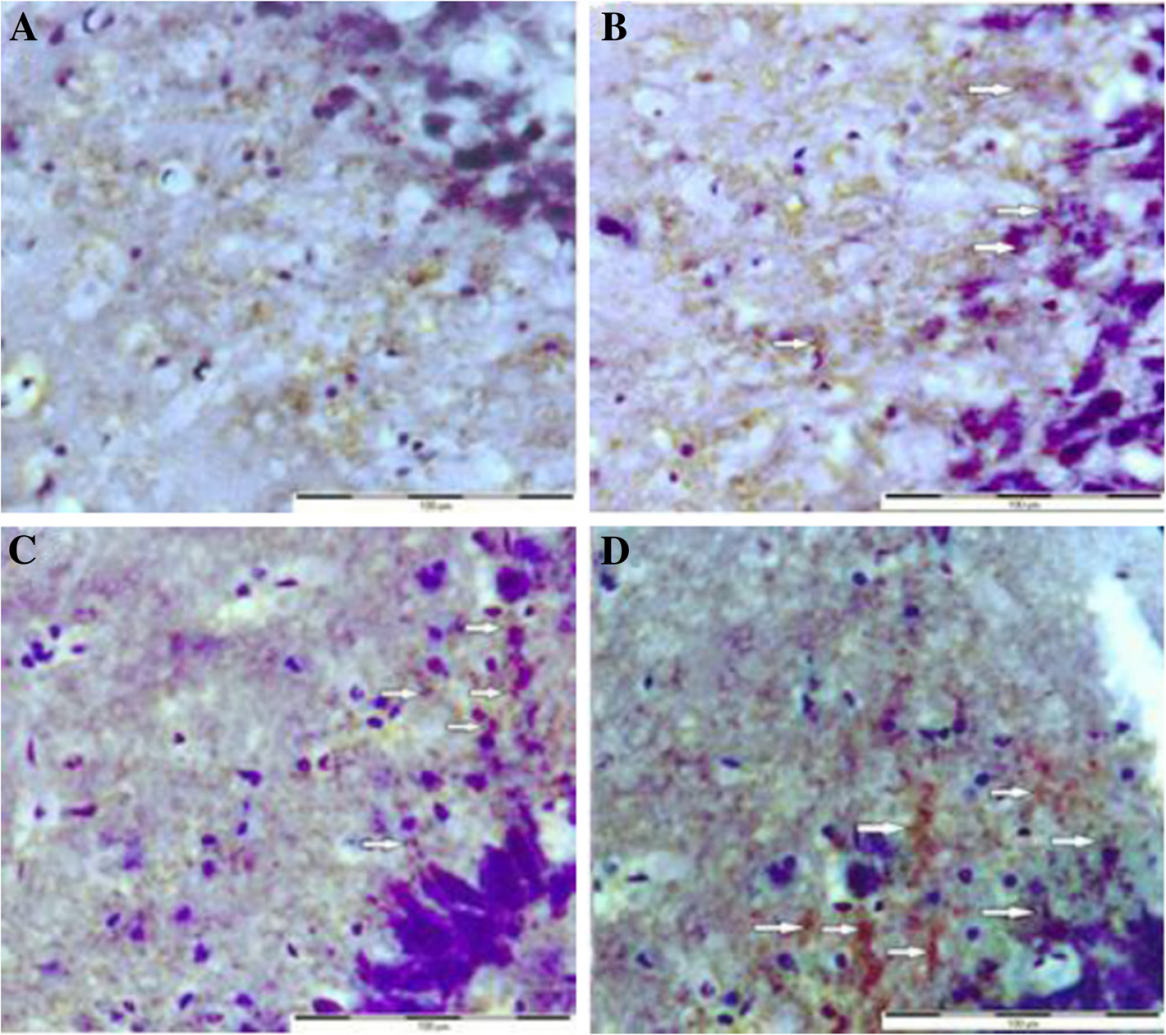
**Light micrograph of the CA1 rat hippocampus.** Note that the intensity of the synaptophysin (arrows) was higher in MMO **(C)** and HMO **(D)** compared to group LMO **(B)** and CTRL **(A)**.

To identify the amount of synaptophysin protein which plays a critical role in synaptic plasticity in the hippocampal CA1 neurons, a immunohistochemistry method was performed on the right hippocampal region of both unsupplemented and supplemented groups. Figure 2 illustrates the effect of dietary fatty acids and different n-6: n-3 FAR on the expression of synaptophysin in the hippocampal region. The intensity of this pre-synaptic protein in the CA1 neurons was increased in animals supplemented with higher n-3 PUFA, i.e. in groups (MMO, HMO) by reducing the n-6: n-3 ratio in the diet. Indeed, the rate of expression of synaptophysin protein can be observed by the change in the intensity of the brown color in micrographs stained with IHC technique. The current results showed that the area of regions with high intensity was 4% in HMO rats. This value for MMO, LMO and CTRL groups was 2%, 0.3% and 0.1%, respectively. Therefore, the HMO and MMO rats which were fed higher amounts of fish oil showed a greater intensity of the brown color compared to the LMO and CTRL groups.

## Discussion

The high hippocampal concentration of total n-3 PUFA in the HMO group observed in this study correlated with the significantly higher levels of n-3 PUFA in this diet. DHA supplied via fish oil dietary was effective in enriching brain tissue fatty acids; the DHA content of HMO group was 20% higher than that of the CTRL group in our study. At the same time, AA decreased below the level of CTRL rats in LMO and HMO fed animals. Our results, concerning the brain balance between DHA and AA induced by dietary n-3 fatty acids, corroborate with some reports [36] which have demonstrated an increase in DHA with a decrease in AA in n-3-rich diet fed animals. The n-3 fatty acid content of the brain is high and has been suggested to be the important factor in brain function as the membrane phospholipid fatty acid composition and configuration of neurotransmitter receptors [37,38].

Memory improvement via PUFA supplementation might be due to the improved membrane fluidity that can affect synaptic plasticity, neurotransmission and synaptogenesis [39]. An increase in the PUFA level will result in fluidization of the neuronal membrane [40]. The neural membrane functions to regulate membrane-bound enzymes, control ionic channel activity, modify the number and affinity of receptors and regulate the production of neurotransmitters which are dependent on the membrane fluidity [40]. Collectively, these could be translated into more efficient signal transduction and sustenance within the entire neuronal network responsible for memory creation and retention. Since the n-3 fatty acids generate changes in membrane fluidity, enzyme activity, and gene expressions, the amount of these fatty acids in the membrane may be a key factor affecting the cognitive and sensitive components, and changing the lipid signaling [40]. The DHA which is the most important n-3 PUFA in neural membranes, is crucial for the maintenance and restoration of neural membrane function and the n-6:n-3 balance in the membrane is important for the neurotransmission and neuroprotection [41].

Our data suggest that the neuron size and number increased in the hippocampus with increasing n-3 PUFA supplementation. In general, the hippocampal neuron size in the HMO rats was larger than those fed lower levels of n-3 PUFA (MMO and LMO). The number of neurons also increased in the supplemented rats. The current results suggest that the neurons in the brains of rats fed the n-3 PUFA supplemented diet developed at a faster rate. These findings are consistent with previous reports by Su [14] on the effects of n-3 PUFA on neurogenesis. The dietary n-3 PUFA can affect the morphological parameters in the hippocampus such as the size of neurons, as previous studies have reported that a deficiency of brain DHA as a source of n-3 PUFA led to a reduction in the neuronal size in the CA1 region of the hippocampus [17]. The increase in the cell body of neurons, likely reflecting an increased synthesis and the concentration of neurofilaments can cause behavioral changes [42]. For example, the increase in cell size and numbers were accompanied by increases in the size of terminal fields and density of boutons [43,44]. Such expansion of the terminals and intensity of vesicle synapses are likely to affect memory formation.

The morphometric changes affecting circuits that convey cortically derived information is critical for hippocampal learning [45]. Memory formation is based on the process of neurotransmission, and one possible explanation of the improvement of neurotransmission may be a consequence of synaptogenesis [13]. Since the neurotransmitter release requires fusion of the synaptic vesicle with the synaptic membrane, a decrease in the membrane fluidity may impair the synaptic transmission [46]. The PUFA with the aid of protein kinase C (PKC) may also contribute to trigger synaptogenesis [47]. Learning and memory formation correlate with modulation of neuronal activity that lead to changes in gene expression and synapse number [48]. Our data showed that the hippocampal neuron size in the HMO rats was larger than the other groups. Previous studies have also reported the decrease in neuron size of the hippocampus in the DHA-deficient diet groups [17]. The decrease in neuronal n-3 PUFA could decrease the nerve growth factor in the hippocampus and might result in a change the hippocampal neuron size [17,24].

The PUFA augment the neurotransmitter ACh formation and release in the brain and the ACh modulates LTP and synaptic plasticity in neuronal circuits that are involved in learning and memory [27]. In the mammalian hippocampus, the LTP is associated with changes in expression of proteins involved in the induction of synaptic plasticity. The supplementation with DHA will increase synaptic plasticity and memory formation through an increase of specific pre- or post-synaptic proteins, which are essential for synaptic plasticity and memory strengthening [49].

In the present study using synaptophysin, a marker of synaptic density and synaptic vesicle formation, it was shown that n-3 PUFA supplementation was able to enhance synaptogenesis in the hippocampus. One possible explanation for the improvement of spatial memory may be a consequence of improved synaptic plasticity and neurotransmission through the enhancement of synaptophysin expression.

The current results showed that PUFA supplementation increased the amount of synaptophysin protein in the hippocampal neurons. Treatment groups with spatial learning improvement displayed increases in the intensity of synaptophysin immunohistochemical staining in the CA1. There is a correlation between behavior and synatophysin intensity in the hippocampus that receives direct cortical inputs [45]. Synaptophysin is the major protein of the synaptic membrane and may play an important role as a channel in synaptic vesicle exocytosis, e.g. in neurotransmitter release. Thus, the early increase in synaptophysin expression may reflect an upregulation of synaptic functions and may be related to the release of the neurotransmitters. Since synaptophysin and other synaptic vesicle proteins have been implicated in the mechanisms of cellular plasticity underlying learning [50], an increase in the expression of this protein might improve memory formation. Synaptophysin is a reliable indicator of synaptic plasticity, and has previously been demonstrated to correlate well with the loss of cognitive function in mouse models with neurodegeneration and in humans with Alzheimer’s disease. The interpretation of these results was based on the observation that these alterations in synaptophysin staining were apparent among the rats with increased spatial learning. In the present study, the effect of increased brain n-3 fatty acids on hippocampus morphology was assessed based on the changes in the amount of presynaptic protein synaptophysin in the CA1 hippocampal neurons. The results indicated that the expression of synaptophysin was increased in the rats supplemented with higher levels of n-3 fatty acids. The PUFA have good protective effects on synaptophysin [51], resulting in increased synaptophisin expression among groups supplemented with increased n-3 PUFA. Since cognitive function is linked to alterations in presynaptic proteins, the increase in synaptophysin may enhance synaptic plasticity leading to the improvement of memory formation.

## Conclusions

In conclusion, this study demonstrated that diets supplemented with higher levels of menhaden fish oil improve spatial memory by incorporating abundant dietary n-3 fatty acids into the membrane phospholipids of the brain. These fatty acids would affect neuronal function by changing the physical properties of the membrane, and influence a variety of physiological membrane functions that depend on the membrane fluidity. This raises the possibility of using natural compounds such as fish oil to improve mental ability such as spatial memory. The alterations in neuronal morphology, biochemistry, and physiology associated with the increased brain n-3 PUFA might lead to the improvement of mental ability and memory.

## Methods

### Animals and diets

Forty individually housed male *Sprague–Dawley* rats weighing 200 ± 20 g were assigned randomly into four treatment groups of ten animals each namely the control group (CTRL), low menhaden oil (LMO), medium menhaden oil (MMO) and high menhaden oil (HMO). After a one week adaptation period, all the rats were fed the experimental diets for 14 weeks. They were maintained under a light/dark cycle 12/12 h at constant temperature (25 ± 2°C) and humidity (50–60%). The experiment was approved by the Institutional Animal Care and Use Committee (IACUC) of the Faculty of Veterinary Medicine, Universiti Putra Malaysia (approval UPM/FPV/PS/3.2.1.551/AUP-R66). All diets were prepared fresh and fed to the animals once daily at 0900 h at 10% of body weight with water available *ad libitum*.

The CTRL, LMO, MMO and HMO groups received standard pellet diets enriched with 7% (w/w) of butter, 0.23% (w/w) fish oil + 6.77% (w/w) soybean oil, 1% (w/w) fish oil + 6% (w/w) soybean oil, and 3.5% (w/w) fish oil + 3.5% (w/w) soybean oil, respectively. The fatty acid composition of treatment diets is presented in Table 4.

**Table 4:** Fatty acid profile of the experimental diets

	**Group**			
**Fatty acid**	**CTRL**	**LMO**	**MMO**	**HMO**
Caprylic Acid (8:0)	0.70	0.10	0.17	0.13
Capric Acid (10:0)	0.87	0.37	0.13	0.09
Lauric Acid (12:0)	6.05	0.06	0.06	0.05
Myristic Acid (14:0)	4.66	1.03	1.26	2.82
Myristoleic Acid (14:1)	0.27	0.07	0.10	0.10
Pentadecanoic Acid (15:0)	0.27	0.14	0.14	0.25
cis Pentadecenoic Acid (15:1)	0.10	0.11	0.05	0.09
Palmitic Acid (16:0)	28.11	14.60	15.16	16.47
Palmitoleic Acid (16:1)	0.84	1.14	1.53	3.93
Stearic Acid (18:0)	6.77	3.96	4.18	4.84
Oleic Acid (18:1n-9)	29.59	30.10	20.35	26.85
Linoleic Acid (18:2n-6)	17.78	40.39	40.37	31.94
α-Linolenic Acid (18:3n-3)	0.28	0.38	0.37	0.27
Arachidic Acid (20:0)	1.51	2.08	2.09	2.39
Arachidonic Acid (20:4n-6)	0.15	0.60	0.13	0.29
Eicosapentaenoic acid (20:5n-3)	0.00	0.61	0.92	3.40
Docosahexaenoic acid (C22:6n-3)	0.00	0.56	0.65	3.59
Total SFA	50.19	24.54	24.43	27.98
Total MUFA	31.54	32.73	33.03	32.29
Total n-3 PUFA	0.28	1.56	1.94	7.26
Total n-6 PUFA	17.99	41.16	40.59	32.47
(n-6): (n-3)	65.09	26.45	22.56	4.47

*CTRL:* control group, *LMO:* low menhaden oil, *MMO:* medium menhaden oil and *HMO:* high menhaden oil.

*SFA:* sum of C8:0, C10, C12:0, C14:0, C15:0, C16:0, C18:0, C20:0.

*MUFA:* sum of C14:1, C15:1, C16:1, C18:1.

Total n-3PUFA: sum of C18:3n-3, C20:5n-3, C22:6n-3.

Total n-6PUFA: sum of C18:2n-6, C20:4n-6.

(n-6): (n-3): Total n-6PUFA (C18:2n-6, C20:4n-6): Total n-3PUFA (C18:3n-3, C20:5n-3, C22:6n-3).

### Fatty acid analysis

Total fat from the experimental diets and brain were extracted according to the methods described by [52] and modified by [53]. The experimental dets approximately 2 g were mixed with of chloroform-methanol (2:1, v/v) containing butylated hydroxytoluene as antioxidant. Then, fatty acids methyl esters (FAME) were preapared using potassium hydroxide and boron trifluoride (BF_3_) (Sigma Chemical Co. St. Louis, Missouri, USA). The FAME were separated by with an Agilent 7890A Series GC system (Agilent Technologies, Palo Alto, CA, USA) using a 30 m × 0.25 mm ID (0.20 μm film thickness) Supelco SP-2330 capillary column (Supelco, Inc., Bellefonte, PA, USA). One microlitre of FAME was injected into the chromatograph, equipped with a split/splitless injector and a flame ionization detector (FID). The injector temperature was programmed at 250°C and the detector temperature was 300°C. The column temperature program initiated runs at 100°C, for 2 min, warmed to 170°C at 10°C/min, held for 2 min, warmed to 200°C at 7.5°C/min, and then held for 20 min to facilitate optimal separation. Identification of fatty acids was carried out by comparing relative FAME peak retention times of samples to standards obtained from Sigma (St. Louis, MO, USA).

### Brain tissue sampling and processing

At the end of the 14-week feeding trial the rats were deeply anesthetized by intraperitoneal injection of ketamine-xylazine (Ketamine 50 mg/kg & Xylazine 10 mg/kg) before the diaphragm muscle was severed. They were then perfused transcardially with cold normal saline (0.9% NaCl), followed by 4% paraformaldehyde in 0.1 m phosphate buffer (PFA, pH 7.4) for 20 min. The whole brain was then removed and post-fixed for 24 h at 4°C. The right hemisphere of the brain was rinsed and transferred to 30% sucrose in 0.1 m phosphate buffer (pH 7.4) at 4°C until saturated. Coronal sections (20 μm) were cut on a cryostat at −22°C and stored at −80°C until immunohistochemistry (IHC) processing. To visualize the morphology of the neurons, the left hemispheres of the brains were dehydrated through graded alcohols and xylene, and embedded in paraffin. Five-micrometer sections were cut and hematoxylin and eosin were used.

The nomenclature and nuclear boundaries used in this study were based on the atlas of Paxinos and Watson [54]. For the dorsal hippocampus, we used sections ranging from −2.5 to −3.5 mm from Bregma [54].

### Morphological measurements: number and size of neurons

Regions of the hippocampus selected for morphological measurements in coronal sections [54] were lying close to the middle of the anterioposterior extent of the brain. Neurons in 10 sections of the hippocampal area were measured from each rat. In each rat, the size and number of neurons in CA1 and CA3 layers at the septum (approximately −2.5 to −3.5 mm from Bregma) in the hippocampus based on the maps of Paxinos and Watson [54] were analyzed. The measuring of the size and number of neurons was performed using ImageJ software (version 1.44p, National Institute of Mental Health, Bethesda, Maryland, USA). The brain of five rats from each group was used and the area of neurons was measured in 10 sections from each rat. The hippocampal neurons were distinguished from the glia on the basis of their size and the presence of a large and relatively pale nuclei and well-defined Nissl material in their cytoplasm. Each neuron was visualized using an oil immersion objective at 100 × magnification.

### Immunohistochemistry (IHC)

For synaptophysin IHC, sections were rinsed in 0.1 m phosphatebuffered saline (pH 7.4), blocked in 0.2% Triton X-100 (TX) and 5% normal donkey serum (Jackson ImmunoResearch Laboratories, Inc., West Grove, PA, USA) for 1 hour at room temperature, and then incubated in monoclonal mouse antisynaptophysin (MAB5258; Chemicon, Temecula, CA, USA), diluted 1: 200 in 0.5% TX and 5% normal donkey serum for overnight at 4°C. This was followed by incubation in biotinylated horse antimouse IgG (1: 2, Vector Laboratories, Burlingame, CA, USA), 0.2% TX and 5% normal donkey serum for 1 h at room temperature and, after rinsing, was incubated with avidin–biotin–peroxidase complex (Vectastain kit; Vector Laboratories, Burlingame, CA, USA) for 1 h at room temperature. The sections were rinsed, reacted with 0.05% 3,3′- diaminobenzidine tetrahydrochloride (Sigma, St Louis, MO, USA) containing 0.01% H_2_O_2_ for 10 min. The sections were rinsed, mounted onto slides, dried, dehydrated and topped with cover slips. Synaptophysin staining was absent in control sections incubated without the primary antibody, confirming that the antibody was synaptophysin-specific.

### Data analysis

The size and number of neurons in the hippocampus were compared across treatment groups using an analysis of variance procedure (ANOVA). Significant different means were then tested using the Tukey (the variances of the groups were equal) and Dunnett’s T3 (the variances of the groups were not equal) post hoc test for analyzing the size and number of neurons, respectively. Data was considered significantly different when *P* < 0.05.

## Competing interests

The authors declare that they have no competing interests.

## Authors’ contributions

TH, TAL and ME conceived and designed the study, participated in data collection and analyses and drafted the manuscript; GYM, MAR, SV participated in the design of the study and drafted the manuscript. All authors read and approved the final manuscript.
